# Automated Protein Turnover Calculations from ^15^N Partial Metabolic Labeling LC/MS Shotgun Proteomics Data

**DOI:** 10.1371/journal.pone.0094692

**Published:** 2014-04-15

**Authors:** David Lyon, Maria Angeles Castillejo, Christiana Staudinger, Wolfram Weckwerth, Stefanie Wienkoop, Volker Egelhofer

**Affiliations:** Department of Ecogenomics and Systems Biology, University of Vienna, Vienna, Austria; UGent/VIB, Belgium

## Abstract

Protein turnover is a well-controlled process in which polypeptides are constantly being degraded and subsequently replaced with newly synthesized copies. Extraction of composite spectral envelopes from complex LC/MS shotgun proteomics data can be a challenging task, due to the inherent complexity of biological samples. With partial metabolic labeling experiments this complexity increases as a result of the emergence of additional isotopic peaks. Automated spectral extraction and subsequent protein turnover calculations enable the analysis of gigabytes of data within minutes, a prerequisite for systems biology high throughput studies. Here we present a fully automated method for protein turnover calculations from shotgun proteomics data. The approach enables the analysis of complex shotgun LC/MS ^15^N partial metabolic labeling experiments. Spectral envelopes of 1419 peptides can be extracted within an hour. The method quantifies turnover by calculating the Relative Isotope Abundance (RIA), which is defined as the ratio between the intensity sum of all heavy (^15^N) to the intensity sum of all light (^14^N) and heavy peaks. To facilitate this process, we have developed a computer program based on our method, which is freely available to download at http://promex.pph.univie.ac.at/protover.

## Introduction

In shotgun proteomics the FCP (Fold Change in Protein) is widely used to compare protein levels of various samples, but neither resolves the dynamics of the proteome in the different biological states that are being compared, nor the mechanisms whereby the system changes from one state to the other [1]–[3]. The elevated abundance of a protein could be the result of an increased synthesis or a decreased degradation rate or a combination of the latter. In recent years, numerous publications employed protein turnover to gain more insight into the regulation of protein abundance [4]–[13]. SILAC-based experimental data can be analyzed with the freely available MaxQuant software for identification and quantification purposes [14]. However, a user-friendly, fully automated, and freely available tool is needed, enabling the extraction of complex partial metabolic labeling data for high throughput studies.

**Figure 1:**
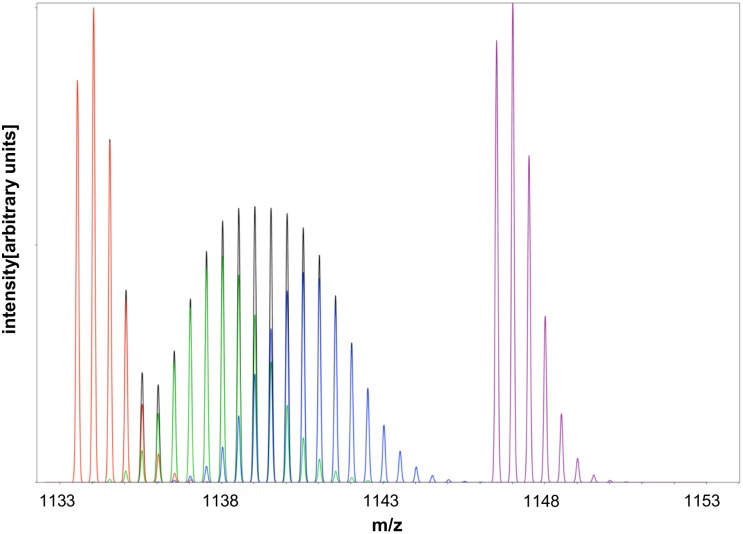
Simulated spectrum of isotopic distribution of the peptide sequence “MPSAVGYQPTLGTEMGTLQER” (charge state 2). The spectrum consists of a peptide species with natural isotopic distribution (red), a peptide with 30% ^15^N incorporation (green), a peptide with 50% ^15^N incorporation (blue), and a peptide with 100% ^15^N incorporation (purple). The sum of all composite spectra is displayed in black.

Since plants are capable of synthesizing their own amino acids, supplying them with an inorganic nitrogen source enriched with ^15^N leads to the incorporation of ^15^N into amino acids and subsequently into fully functional proteins. The higher the degree of ^15^N incorporation, the higher the mass shift of the resulting mass spectrum. Full incorporation of ^15^N results in a mass shift of all isotopic peaks compared to the ^14^N form of the peptide (see purple spectrum in Figure 1). In the latter spectrum, there are still isotopic peaks present, mainly due to the contribution of ^13^C. A vast number of combinatorial possibilities of isotopomers and isotopologues range from the light ^14^N to the pure ^15^N form, known as partially labeled peptides. The resulting mass spectra of individual proteolytic peptides are a composite of all peptide species of variable ^15^N incorporation (see also example Figure 1). This adds to the inherent complexity of biological shotgun-proteomics samples, due to the increased isotopic envelope of individual spectra. Therefore, the main objective of this work was to develop an efficient algorithm for fully automated protein turnover calculations, which can be applied to any kind of sample data arising from partial metabolic ^15^N labeling experiments, no matter the type of organism or tissue.

Software tools coping with partial metabolic labeling data in an automated fashion already exist. Commercial in conjunction with freely available software were used to analyze mammalian pulse chase LC/MS data [15]–[17]. The latter method relies on a combination of ^14^N and ^15^N spectral counts with MS1 information, and requires every peptide quantitation event to have an associated ^15^N MS2 peptide identification [15]. Thus, fully ^15^N labeled peptide species are essential, in contrast to the method presented within this manuscript, which aims to analyze partially ^15^N labeled peptides.

The software “ProTurnyzer” introduced by [18] is available upon request. It accepts pep.xml files in conjunction with RAW data files from Thermo Scientific. Each RAW file (LC/MS file) depends on one corresponding pep.xml file containing the peptide sequence and retention time information necessary to extract data from the RAW files. This means that every RAW file is used for peptide identification purposes as well as for protein turnover analysis. Accordingly, each LC/MS measurement has to be subjected to database dependent identification. Since the vast majority of shotgun proteomics search engines rely on the MS2 spectra of monoisotopic precursors for identification purposes, this approach is only applicable for very low partial metabolic labeling rates.

Another software processing ^15^N partial metabolic labeling data, named “TurnStile” [9], is available upon request. The program uses centroided mzXML and MS Excel files (providing peptide sequence, charge state, and retention time start and end information) to extract spectral envelopes. Subsequently, multiple spectra are averaged and fitted in order to derive the ^15^N incorporation percentage and intensities of the light and heavy isotopic envelopes. Retention time values can be adapted for each file individually.

Both “ProTurnyzer” and “TurnStile” process each LC/MS file individually, average over multiple spectra (producing a single averaged spectrum for the extraction of the spectral envelope of each peptide) and subsequently fit experimental data to theoretical values. One of the main differences between “TurnStile” and “ProTurnyzer” is how they calculate the averaged spectra. “TurnStile” averages over all scans within a given retention time start and end point, and have applied a 3 min window for their data [9]. In contrast, “ProTurnyzer” extracts peak intensities from RAW MS1 scans within an elution time window of 60 s before and after the corresponding MS2 scan, by summing up all intensities bound by local minima surrounding the maximum within 20 ppm [18].

The presented method is fundamentally different to the previously mentioned methods, since all LC/MS files of a time series experiment are processed together, in reverse chronological order (from the maximally to the minimally labeled state). The basic idea behind this approach is the assumption that the spectral envelope of the maximally labeled Time Point will always have the maximum number and intensity of isotopic peaks, given that the monoisotopic precursor is still present. This leads to the best signal to noise ratio for the isotopic peaks. The peak picking of every Time Point depends on the previous one. Thus, an interdependency of Time Points is established, that reduces picking of noise. The application expects centroid or profile mode mzML files in conjunction with a text file containing peptide identification information. This algorithm has been implemented in a program, written in Python, which is freely available to the scientific community at http://promex.pph.univie.ac.at/protover.

## Methods

Since we cannot assume that every protein will be present in the sample at any given time (or present in a detectable quantity), the question remains for which proteins/peptides to look in a partial metabolic labeling LC/MS shotgun proteomics data set, if this data cannot be used for peptide identification. We have circumvented this problem with the experimental design of our study. Parallel to a ^15^N labeled sample group, we have grown a set of ^14^N control plants. The LC/MS data generated from samples of the latter group was used for peptide identification (for details see Document S1). Seven-week-old *M. truncatula* plants were split into two groups a control (non-labeled) and a treatment (fertilized with ^15^N enriched ammonium nitrate) group. Samples were taken for five consecutive days. After protein extraction and digestion, the samples were analyzed by LC/MS. Since the incorporation of ^15^N leads to fully functional proteins, we assumed a very similar protein composition for the control and the treated sample groups. Subsequently, the control group was used for peptide identification, generating a list of peptide sequences, their corresponding charge state and retention time as well as the accession number of the inferred protein [19]. This list together with the samples of the treatment served as the input for the program at hand (for a more detailed description see Document S1).

The current software does not require unlabeled data/samples in any way. We’ve employed an experimental design which includes control samples that are unlabeled in order to use these unlabeled samples for peptide/protein identification, which in turn serves as the input for the program. If previously identified peptide sequences, charge states and retention times are known, this unlabeled control is unnecessary. Any partially labeled ^15^N LC/MS shotgun proteomics data could be evaluated with this software.

### Method Outline

The following steps were used in our algorithm:

- Sort the input files in reverse chronological order
- Calculate the isotopic peaks (isotopic envelope) for a given peptide sequence and charge
- Pick peaks according to template
- Filter out co-eluted picked peaks
- Choose best scan within retention time-range
- Set new template from experimental data for the next file
- Filter noise at TP_0_ (first Time Point)
- Calculate the RIA (Relative Isotope Abundance)
- Post processing filter
- Data export
- Compatibility

### Sort Input Files in Reverse Chronological Order

Since partial metabolic labeling experiments consist of time course measurements, regardless of pulse-chase or other experimental designs, the chronological order of the measurements can be taken into consideration. In our approach we search the files in reverse chronological order (from the maximally labeled to the minimally/non-labeled). The number of peaks of the isotopic envelope increase with time, as more ^15^N is incorporated, therefore decreasing when reversing the order. The first file (TP_MAX_) is searched with a template of theoretically calculated m/z values for a given peptide, producing the picked peaks of the measured spectrum. The template for the next Time Point (TP) consists of only those peaks that could be picked for the previous TP. The extracted (experimental) spectrum serves as the template (replacing the number and position of the peaks in the template, but not their theoretically calculated value). Thus the m/z values do not change, but the number of values in the template changes dependent on how many of them were found in the previous TP. This leads to the next extracted spectrum which is again used to extract the spectrum of the next file (TP_MAX_-2). This approach enables the algorithm to never pick more peaks than in the previous time point, which in turn reflects the biology of the underlying data.

### Calculate the Isotopic Peaks (Isotopic Envelope) for a Given Peptide Sequence and Charge

The possible isotopic envelope and thus the peaks for the theoretical template are calculated as follows: For each peptide sequence, the sum of its individual C, H, O, N, S atoms is built and multiplied with the mass of its most abundant isotope. This produces the monoisotopic peak. All subsequent peaks are calculated by exchanging the mass of a ^14^N by a ^15^N atom. The largest isotopic peak is the ^15^N monoisotopic peak. Thus, the template consists of as many peaks as there are nitrogen atoms plus one (n+1). Finally, the mass values are converted to m/z values by the addition of as many protons as charges, divided by the number of charges.

### Pick Peaks According to Template

For any given peptide, the mzML file is searched within a user defined retention time window, allowing for common retention time deviations occurring in Liquid Chromatography (LC). Every Full Scan within this window is processed as follows:

The most abundant m/z value is picked within a user-defined range (e.g. +/−10 ppm) of the monoisotopic peak.
The algorithm only searches for subsequent peaks if the first peak (the monoisotopic peak) was found.
All subsequent peaks are picked analogously (since the mass accuracy decreases with decreasing intensity, this value can also be adjusted separately by the user dependent on the given data).

### Filter Out Co-eluted Picked Peaks

**Figure 2:**
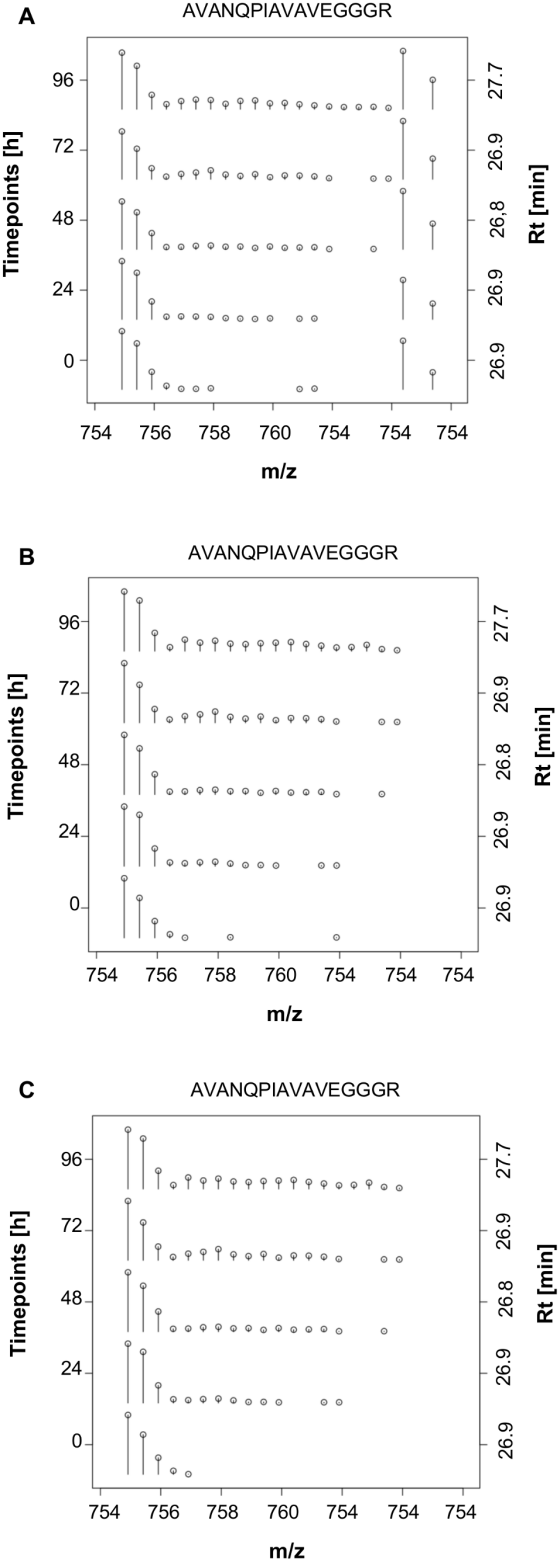
Picked peaks of the peptide sequence “AVANQPIAVAVEGGGR” at all Time Points (TP). The abscissa indicates the mass to charge ratio. Left ordinate indicates Time Points (corresponding to the user-given number in the “Experiment file”), right ordinate indicates the retention time (in minutes) of the scan used to pick the peaks. The individual spectra are normalized to the base peak of the given spectrum. A: Without the application of any filters. B: Filter out co-eluted picked peaks. C: Filter out co-eluted picked peaks and Filter noise at TP_0_.

In order to remove overlapping peaks belonging to another peptide, the following filter was implemented. If the ratio of the current peak is 3 times higher to the preceding peak (empirically found value), the current peak is removed from the raw data. Subsequently, the appropriate peak is picked again. This routine of removal and re-picking is iterated either until no more peaks are removed from the raw data, or no more peaks remain to be picked from the raw data (see Figure 2.A and 2.B).

Furthermore, the application of the co-eluted picked peaks filter in conjunction with the penalty of the total score addresses the issue of complex overlapping envelopes.

### Choose Best Scan within Retention Time-range

A single scan is used for peak picking, and not a scan as a result of averaging over multiple scans. The latter could potentially lead to an increase in noise and or elevate the complexity of the spectrum, since analytes eluting with similar retention times are prone to produce overlapping isotopic envelopes, especially for partial metabolic labeling data. In order to choose the best retention time (scan) from within the given retention time range and to evaluate the quality of the selected data points for a given peptide, a total score (TS) is calculated for each scan. The maximum score is selected and the corresponding data points saved
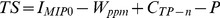
(I)


The total score is composed of the following components:

•**I** ***_MIP0_***: logarithm to the base 10 of the intensity of the Monoisotopic Precursor (MIP0) in arbitrary units.

•**W** ***_ppm_*** **:** Weighted sum of ppm deviations of a given peptide spectrum.

**(II)** 
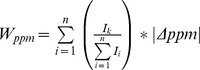
, with

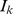
: Intensity of a peak in the given peptide spectrum (in arbitrary units).

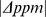
: The absolute value of the ppm deviation of the m/z value of compared to the theoretically calculated m/z value.

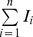
: Sum of all peak intensities in the given peptide spectrum (in arbitrary units).

•**Coverage: (III)** 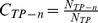

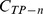
: Coverage of a given Time Point (TP) with n from Zero to the maximum number of Time Points, in reverse chronological order.

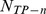
: Number of picked peaks of the experimental spectrum of the previous Time Point, respectively number of theoretically calculated peaks of a given peptide sequence for last Time Point (TP_MAX_).

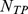
: Number of picked peaks of the experimental spectrum of the given Time Point.

•**Penalty: (IV)**

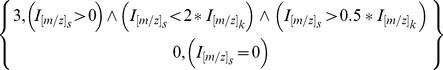
with: 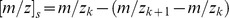.

If the first peak of the experimental spectrum is an isotopic peak of another peptide a penalty is applied. First, the mass difference 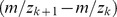
of the first to the second peak is calculated. This value is deducted from the first peak. Within a range of +/−10 ppm of *m/z* the peak with the highest intensity is selected. If the intensity of this peak is higher than half and less than twice of the intensity of **I** ***_MIP0_***, then a penalty of 3 is applied.

### Set New Template from Experimental Data for the Next File

After the processing of the initial file (TP_MAX_), the template changed from all theoretically calculated peaks to only those peaks that could be picked for the previous TP. If not a single peak could be picked in the “previous round”, all theoretically calculated values remain as the template.

### Filter Noise at TP_0_ (First Time Point)

The spectrum of the minimally labeled measurement is used to determine which peaks represent the ^14^N peptide (natural abundance of N). In order to remove data points that are rather considered noise than low abundant peaks, all data points following a missing peak are removed from the spectrum (from low to high m/z-values, see Figures 2.B and 2.C).

### Calculate the RIA (Relative Isotope Abundance)

The Relative Isotope Abundance (RIA) is defined as the ratio of the ^15^N to all isotopic peaks [1], [20]. Since no ^15^N incorporation has taken place at the very first measurement, all peaks present in TP_0_ are considered part of the ^14^N peptide species. For each individual experimental spectrum the RIA is calculated as follows:
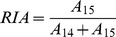
(V)with

A_14_: Sum of intensities of all ^14^N peaks (natural abundance).
A_15_: Sum of intensities of all ^15^N peaks (isotopically labeled).

In order to differentiate the natural abundance from the enriched part of an overlapping isotopic peak (see Figure 1 red and green species overlapping at e.g. the 5^th^ isotopic peak), the relative intensity values at TP_0_ are taken into account when calculating A_14_ and A_15_ for all other TPs. (for more details please see Document S1).

### Post-processing Filter

**Figure 3:**
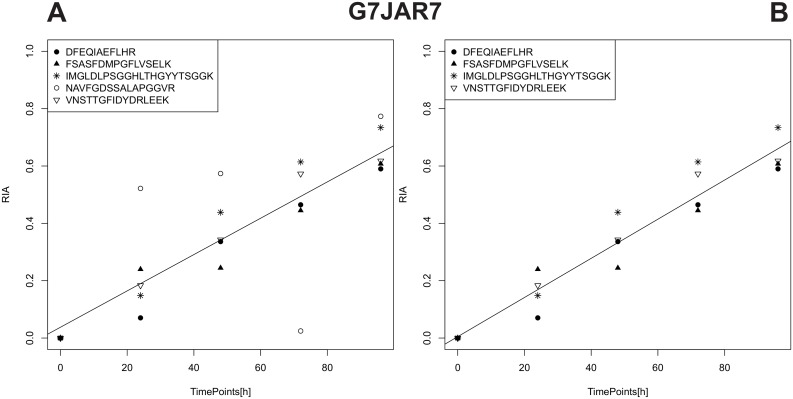
Relative Isotope Abundance (RIA) plots. The abscissa represents the Time Points (provided by the user in the Experiment File) and the ordinate the RIA ratio at the given Time Point. The titles of the plots indicate the Accession Number for the given data. The legend shows all peptide sequences that could be attributed to the given protein. A: illustrates the RIA plot for G7JAR7 without the application of any filters. B: RIA plot for G7JAR7 with the application of post-processing filter.

**Figure 4:**
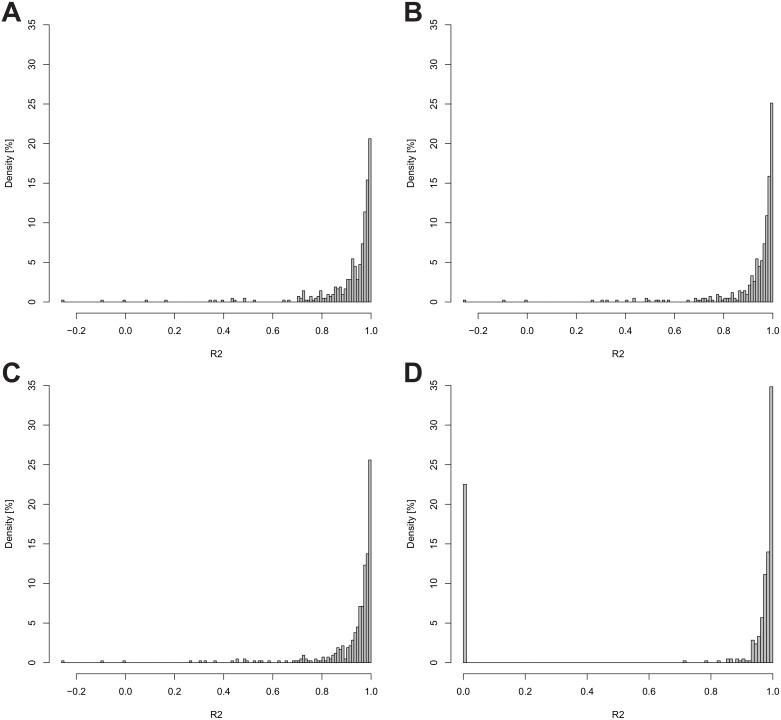
Histograms of the regression coefficient versus the density of proteins. Histograms of the regression coefficient versus the density of proteins, comparing no filter (A), co-eluted picked peaks filter (B), co-eluted picked peaks and filter noise at TP_0_ (C), and all filters combined (D). (A-D) with 422 cases each. For each Time Point, all peptide RIA values (associated with an Accession Number) were averaged. Subsequently the linear regression was calculated, and thereof Histograms produced. (D) includes (the 94 of the 422) proteins that were removed by the post-processing filter.

Due to the incorporation of ^15^N, novel synthesis of a given protein will produce an increase of the A_15_ term when measuring its proteolytic peptides. We assume that the RIA for a given protein will stay constant or increase with time due to the following reasons. A fictitious protein without novel synthesis, but with degradation, would produce a constant A_15_ and a decreasing A_14_ term (see formula (V)), and thus a constant numerator and a decreasing denominator with time, leading to an increase of the RIA over time. A fictitious protein with novel synthesis, but without degradation, would produce an increasing A_15_ and a constant A_14_ term, also leading to an increase of the RIA over time. Furthermore, a protein with novel synthesis and degradation will always produce an increasing RIA over time. Therefore, a post-processing filter was devised, removing all peptides whose RIA decreased over time (see Figure 3.A and 3.B as well as Figure 4). If data for one Time Point of a peptide is missing, the RIA for that Time Point is not calculated. Subsequently, this peptide will not pass the post processing filter even if all other Time Points produced a positive result.

This very stringent filter reduces the data to the most stable signals that can be traced throughout the entire data set (see Figure 4.D). Please see Document S1 for configuration options (Post-processing configuration options).

### Data Export

In order to save the output of the data analysis, the extracted spectra and useful additional information is saved to tab delimited txt files for easy import into Excel (see Document S1). Additionally, two-dimensional plots of peptide spectra for all the Time Points can be plotted as pdf files (see example Figure 2.C). The RIA for each protein can be plotted as well (see example Figure 3.A and 3.B without the regression line).

### Compatibility

Furthermore, the presented program runs on all commonly used operating systems (Windows, OSX, and Linux), is independent of the tissue being analyzed, and is not restricted to any specific type mass spectrometric data.

## Results and Discussion

Protein turnover experiments are most often performed using cell cultures of human or plant cell lines. The uniformity of the given cell type and the possibility to quickly exchange the growth medium enable full incorporation of heavy labels within hours or at maximum a couple of days [1], [3], [8], [13]. The experimental design of the present study is inherently different, due to the fact that entire plants were grown in pots to their fully functioning potential, closely resembling the phenotype of the species in the wilderness of nature. This results in dramatically reduced measurable turnover rates due to the following reasons: The exchange of the light by the heavy amino acid pool cannot be performed by simple plating (as in cell cultures), but by supplying an inorganic nitrogen source that has to be taken up by the roots and incorporated into amino acids and subsequently into proteins, in contrast to SILAC experiments [21] where fully labeled amino acids are provided in excess, and plant cell cultures, where a labeled nitrogen source replaces the unlabeled form immediately. The degradation of existing light or marginally labeled proteins feeds the light amino acid pool, thereby counteracting the relative increase of the heavy amino acids. In order to ensure full labeling, plants were grown with ^15^N medium for over 12 weeks [22]. Therefore, the RIA values of the data set utilized within this study are generally low, but much closer to in situ-growth conditions. After 5 days of labeling, the mean of all RIAs is still below 50% (data not shown). The higher the intensity of the signal, the higher the mass accuracy and vice versa. Manual inspection of the extracted spectra and comparison with the raw data showed that highly abundant peptides lead to fewer missing peaks as well as to congruence of the resulting RIAs, while low abundant peptides showed higher variability, since true positive peaks might not fall within the calculated mass range, but random noise could. The three biological replicates of the test data set showed that over 800 peptides of the 1419 identified peptides passed all three previously described filters, with a variability that can be expected of independent biological replicates. Naturally, the quality of the extracted spectra and thus the output strongly depends on the quality of the input data. Measuring the LC/MS data with a high mass resolution is beneficial, since overlapping peaks are more likely to be resolved and thus enable the algorithm to pick the proper peaks. Instability or poor ESI-spray quality can lead to missing or noisy spectra and reduce mass accuracy. Peptides with missing spectra at any given Time Point will eventually fail to pass the filters. The major steps of the algorithm will be discussed as follows.

### Performance of the Applied Filter

The effect of removing co-eluted picked peaks filter as described in Methods becomes apparent when comparing Figure 2.A to 2.B, as the two peaks with the highest m/z-values were excluded from the spectrum. The effect of filter out noise at the first Time Point (TP_0_) is visible when inspecting the extracted spectrum of Figure 2.C compared to 2.A or 2.B, as all peaks following an empty position (missing peak) are removed from the spectrum. The ameliorated peak picking of spectral envelopes of peptides, due to the incorporation of the latter two filters, not only affects the extracted spectra, but also the resulting RIA of the associated proteins. The post-processing filter, described in the Methods part, removes the peptide sequence “NAVFGDSSALAPGGVR” (hollow circle as symbol) (see Figure 3.A and B), due to the lack of an increasing RIA over time. Linear regression of mean RIA values per Time Point, yielded an increase in the regression coefficient from 0.978 (Figure 3.A) to 0.997 (Figure 3.B). Only the application of the co-eluting picked peaks filter affects the total score (lowers the coverage term) and thus potentially alters which scan is chosen for spectral extraction.

The variability of the calculated RIAs for a protein decreases when applying the previously described filters. The overall effects of the various filters are illustrated in Figure 4.A to D. For each protein, all associated peptide RIAs were averaged for each Time Point and a linear regression calculated. The density distribution of regression coefficients (R^2^) of all 422 proteins with and without the application of the previously described filters are shown in Figure 4. The fraction of high R^2^ values increases with the application of the filters. Since the post-processing filter removes peptides, all subsequently removed protein R^2^ values were set to Zero (see Figure 4.D). The fraction of proteins with a regression coefficient between 0.95 and 1.0 starts at 59%, without the application of any filters, increases to 64%, with the application of the co-eluted picked peaks filter, increases further to 66% with the additional application of the filter noise at TP_0_ filter, and finally reaches 89% with the additional application of the post-processing filter (due to the removal of values). The increase in precision of the RIA values after application of the filters is corroborated by the change in the regression coefficients.

**Figure 5:**
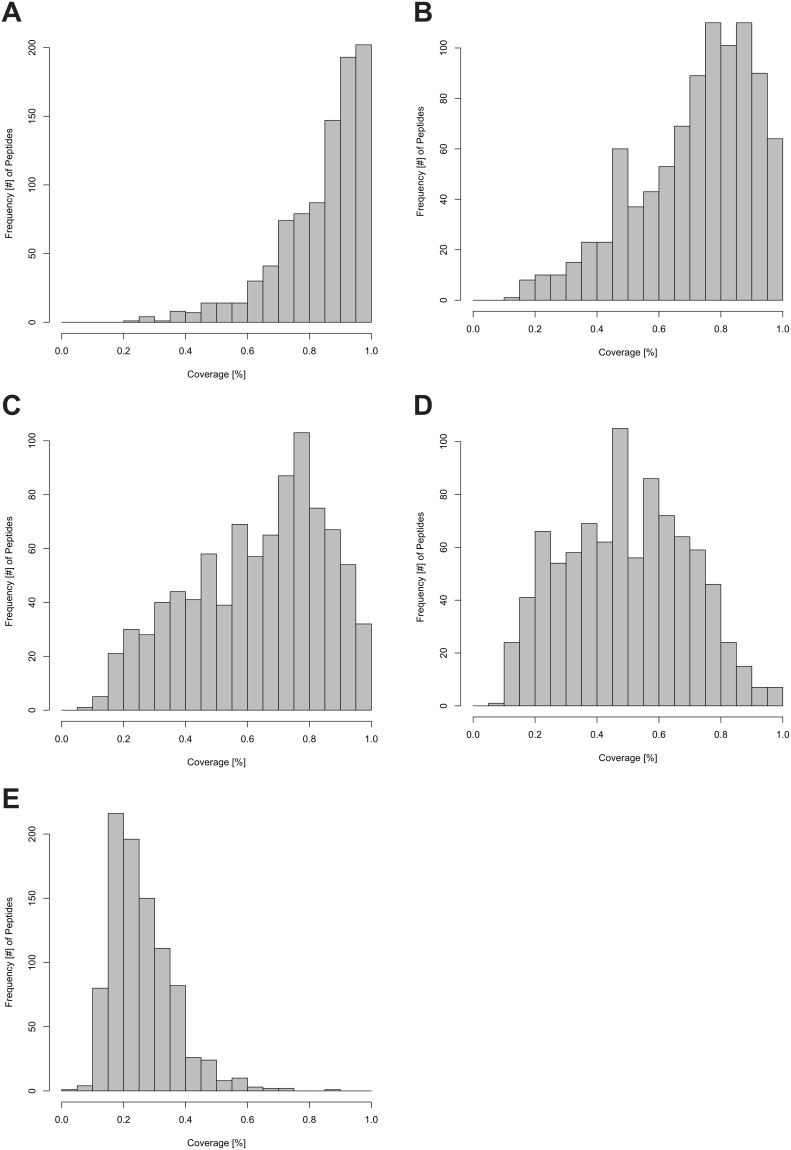
Histogram plots of the coverage versus frequency of peptides. The abscissa represents the coverage in percent. The coverage is calculated analogous to formula (III) the sole difference being the constant denominator N_MAX_. Frequency of peptides indicates the number of peptide sequences with a given coverage (number of cases 1419). All five Time Points (from A to E, of one biological replicate) from 96 h (A) to 0 h (E), in 24 h intervals of ^15^N incorporation are shown.

Each histogram shown in Figure 5 displays the frequency as a function of the coverage of peptides at a given Time Point. Generally, when comparing the histograms in a reverse chronological order (from maximum time point (TP_MAX_) to minimum time point (TP_MIN_), 96 h to 0 h) and thus from the maximally labeled to the minimally labeled state, a trend from a negative skew to a positive skew with intermediate stages can be observed (see Figure 5. A to E). The distribution at TP_MAX_ clearly shows a high coverage for most of the peptides and as the coverage decreases so does the number of peptides. This reflects the underlying biology of the experimental setup showing the partial labeling state of the proteins and thus of their proteolytic peptides. Due to the varying turnover rates, the coverage cannot be constant for all 1419 cases (peptides) at any given time (it should however be constant for all peptides associated with a protein at any given time). As described in the Methods (see “Set new template from experimental data for the next file”), the algorithm was trained to produce a decreasing coverage over time. Figure 5 illustrates the results of the implementation of this desired functionality.

Performance of the developed strategy is demonstrated by the automated protein turnover calculations of 1419 peptides from five Time Points (n = 3, three biological replicates).

Many studies express protein turnover as % turnover per hour (log ratio of heavy to light per hour for SILAC experiments show linear correlation). We are dealing with an entire organism, not a specific cell type, thus we would not expect the synthesis and degradation rates to be constant over time, but rather showing distinct biologically relevant and interesting dynamic kinetics.

### Biological Applicability

The amount of information that can be generated with the presented automated method, is very high, specifically due to the coupling of partial metabolic labeling with high throughput shotgun proteomics, in contrast to the excision of proteins from gel spots [22], [23]. Within the given dataset, the Glycine-rich RNA binding protein (Uniprot accession number: G7JG67) showed a high turnover rate (RIA) in all biological replicates (0.716 mean +/−0.01 standard deviation of 3 biological replicates at TP_MAX_). The protein plays a functional role in processing, transport, localization, translation and stability of mRNAs and the high turnover rates are in accordance to previous plant protein turnover measurements [23], [24]. In contrast, a low protein turnover rate (RIA) was observed for the Harpin binding protein containing a conserved fibrillin domain (Uniprot accession number: G7I4U4) (0.398 mean +/−0.021 standard deviation of 3 biological replicates at TP_MAX_). Plant fibrillins expression increases during acclimation to various biotic and abiotic stresses (reviewed by [25]). The observed low RIA after five days of ^15^N metabolic labeling is in line with the assumption of low stresses during the experimental period.

### Comparison to Other Approaches

The presented algorithm is based on data analysis in reverse chronological order (a unique and novel feature), and doesn’t subsequently fit data to theoretical relative isotope abundances, but uses experimentally derived intensity values for subsequent RIA calculations. Assuming that the monoisotopic precursor is still present, the spectral envelope of the maximally labeled Time Point will always have the maximum number and intensity of isotopic peaks, leading to the best signal to noise ratio. An interdependency of Time Points is established that reduces picking of noise, since the peak picking of every Time Point depends on the previous one.

The presented algorithm is trained to pick the best possible scan within the user-given retention time range, enabling large retention time deviations that can occur in high throughput studies. Switching (renewing) liquid chromatography columns (sometimes done between batches of samples), often leads to retention time shifts. A major strength of our approach is that it can cope very well with these shifts. The user has to simply set a higher retention time range in the „experiment-file“, which will lead to a prolonged runtime (more data has to be processed). The algorithm will still pick the correct scan, making high-throughput studies feasible. In contrast, “TurnStile” [9] averages over a user-defined retention time range, thus potentially averaging over isobaric or isomeric peaks or even noise not belonging to the target. In order to circumvent this behavior, the user would need to set a very narrow retention time range and potentially adapt this setting for each individual file, leading to an enormous work-load contradicting the computational automation of the workflow and impeding high-throughput data analysis (see Figure S1).

Comparing the calculated RIA values, the protein with the Uniprot accession number G7IF28, with 9 associated peptides, displays a low protein turnover when applying our approach (RIA values ranging from 0.0 to 0.35, and linear regression coefficient (R^2^) of mean RIA values is 0.998, see Figure S2.A and S2.B). In contrast, the output generated with “TurnStile” displayed a spread of data, the resulting RIA values reach from 0.23 to 0.99, encompassing a large part of the range of possible values (with R^2^ =  −0.2213). The protein with the accession number G7JG67, with 5 associated peptides, displays a high protein turnover when applying our approach, with a linear regression coefficient of R^2^ = 0.992 (RIA values ranging from 0 to 0.76). Except for the last two Time Points the RIA values of the peptides at a given Time Point derived from “TurnStile” analysis are neither similar nor do they indicate a trend towards an increase in RIA over time (with R^2^ =  −0.568, and the spread of the data reaches from 0.09 to 0.85 for the RIA) (see Figure S2.C and S2.D). For further comparison see Document S1.

### Computation Rate

The computation rate depends on the amount of LC/MS data, the number of identified peptides, and the user-defined retention time range. The runtime increases linearly as a function of the retention time range and/or the number of identified peptides. E.g. given five mzML-files with about 5 GB of data, 1419 identified peptides, and 2 min retention time range, the runtime was about 40 min. Principally, many files can be processed with the given program (it was tested with about 60 GB of data). One strength of the algorithm is to pick the proper scan despite isobaric peptides in the chromatographic domain. Therefore, using a high retention time range is recommended despite the extended runtime.

### Outlook

Implementation of a Graphical User Interface (GUI).
Post-Translational Modification (PTM) support.
Differential data analysis of treatment groups with repect to biological and technical replicates.

## Supporting Information

Figure S1 **TurnStile output strongly depends on Rt range.** The abscissa represents the Time Points and the ordinate the RIA ratio at the given Time Point. The titles of the plots indicate the Accession Number for the given data as well as the retention time window used for data analysis. From Ai to Aii to Aiii (note: the legend for these sub-plots shown at the bottom) and from Bi to Bii to Biii (note: the legend for these sub-plots shown at the bottom) the retention time window decreases from 10 min to 90 s to individually adapted values for every peptide for every file (in the range of 15 to 45 seconds).
(TIF)

Figure S2 **Qualitative comparison of TurnStile vs. Protover.** The abscissa represents the Time Points and the ordinate the RIA ratio at the given Time Point. The titles of the plots indicate the Accession Number for the given data. The legends show all peptide sequences that could be attributed to the given protein. A and B show the RIA plots for G7IF28 (note: the legend for both sub-plots only shown in the right sub-plot). C and D show the RIA for G7JG67 (note: the legend for both sub-plots only shown in the right sub-plot). The data illustrated in A and D were processed using TurnStile with a 90 s retention time window (the recommended setting). B and D were processed using Protover with a 10 min retention time window (+/−5 min) (the recommended setting).
(TIF)

Document S1 **Supplements.** Detailed description of experimental procedures and methods, data analysis and comparison with other algorithm. Calculation of relative RIA, discussion of averaging scans as well as comparison of Retention Time settings.
(DOC)
