# Supplementary figures and images for: Automated Protein Turnover Calculations from 15N Partial Metabolic Labeling LC/MS Shotgun Proteomics Data
Source: PLoS One. 2014 Apr 15;9(4):e94692. doi: 10.1371/journal.pone.0094692 (PMC3988089; doi:10.1371/journal.pone.0094692)

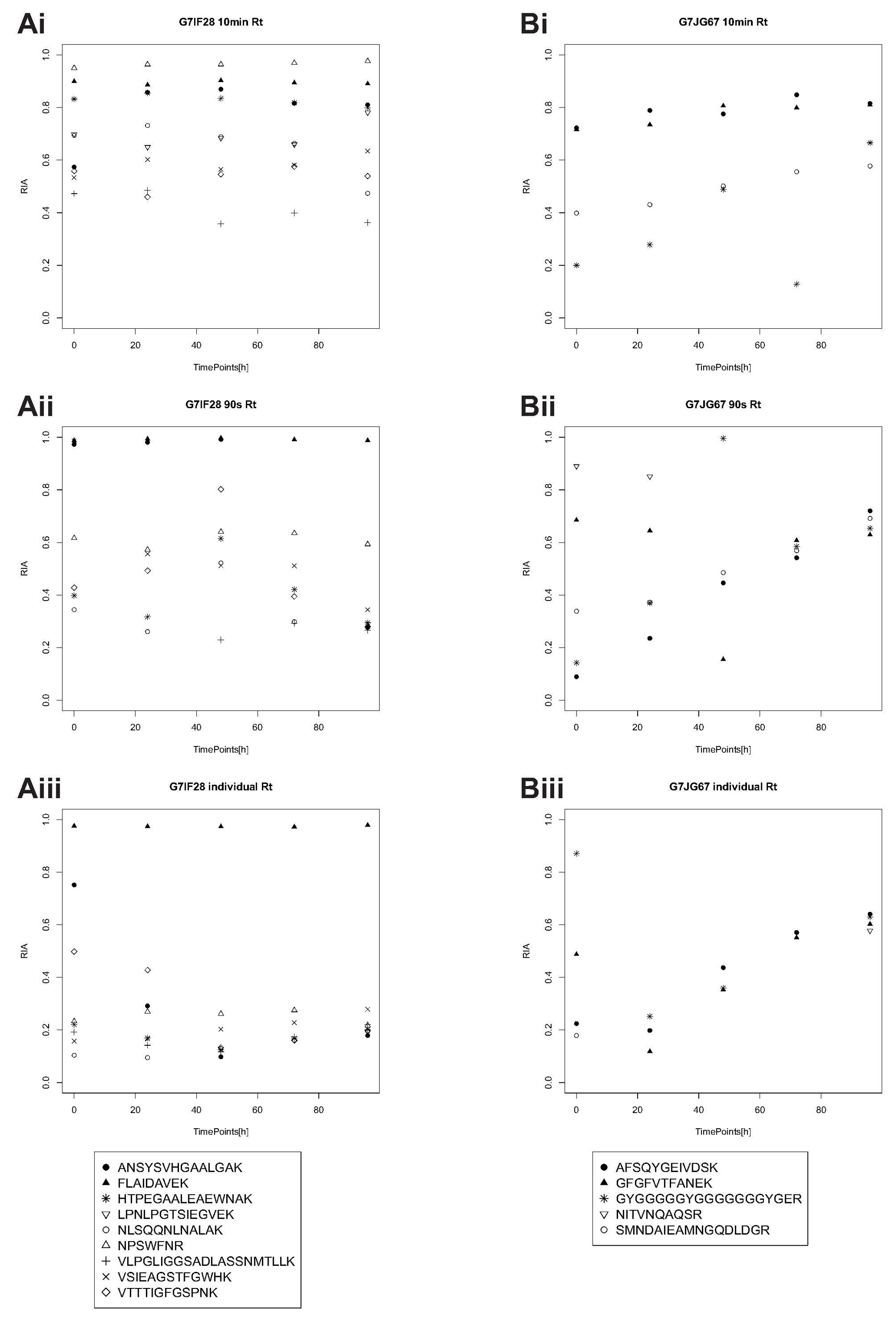

Supplement: Figure S1 — TurnStile output strongly depends on Rt range. The abscissa represents the Time Points and the ordinate the RIA ratio at the given Time Point. The titles of the plots indicate the Accession Number for the given data as well as the retention time window used for data analysis. From Ai to Aii to Aiii (note: the legend for these sub-plots shown at the bottom) and from Bi to Bii to Biii (note: the legend for these sub-plots shown at the bottom) the retention time window decreases from 10 min to 90 s to individually adapted values for every peptide for every file (in the range of 15 to 45 seconds). (TIF) [file pone.0094692.s001.tif]

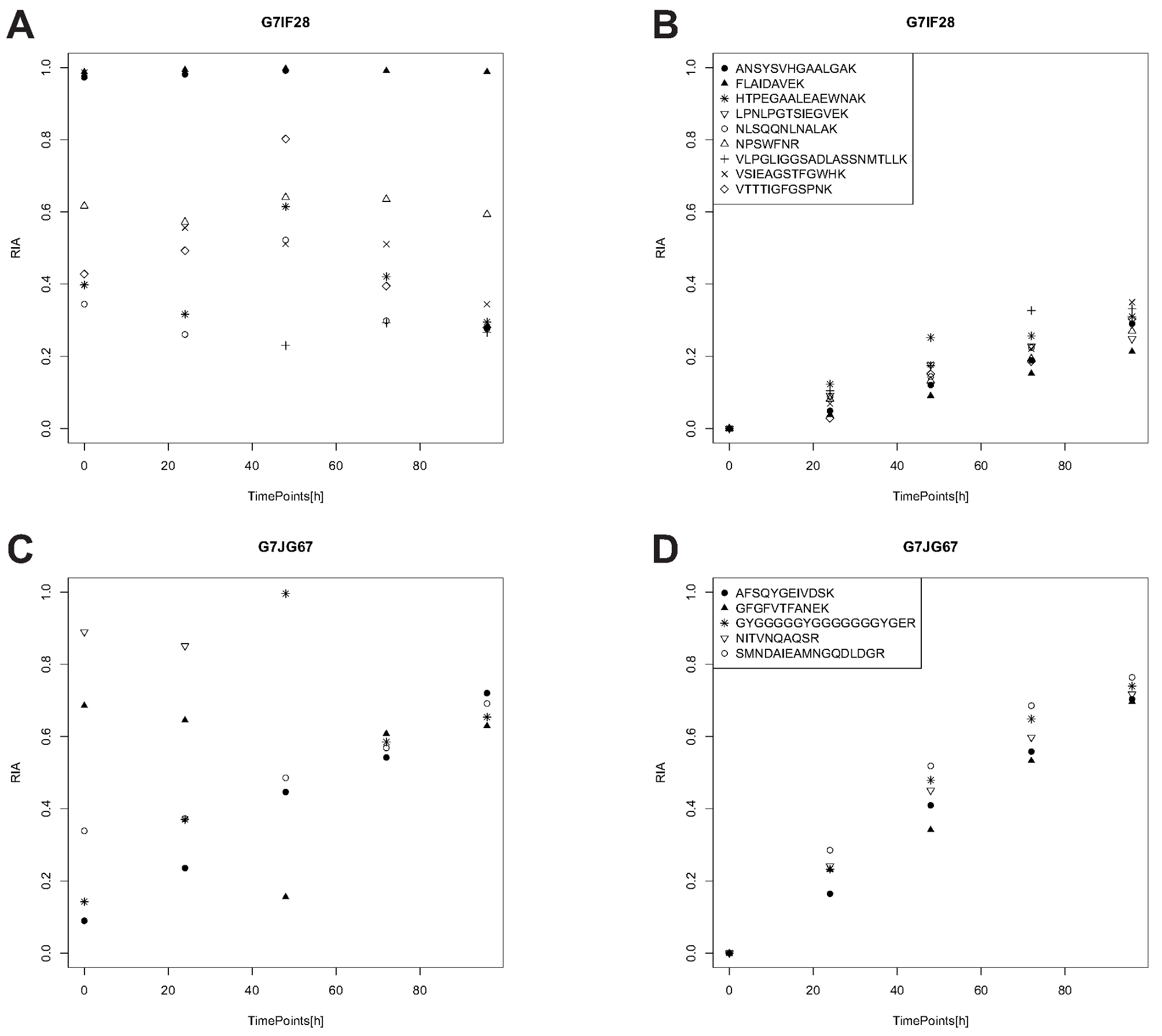

Supplement: Figure S2 — Qualitative comparison of TurnStile vs. Protover. The abscissa represents the Time Points and the ordinate the RIA ratio at the given Time Point. The titles of the plots indicate the Accession Number for the given data. The legends show all peptide sequences that could be attributed to the given protein. A and B show the RIA plots for G7IF28 (note: the legend for both sub-plots only shown in the right sub-plot). C and D show the RIA for G7JG67 (note: the legend for both sub-plots only shown in the right sub-plot). The data illustrated in A and D were processed using TurnStile with a 90 s retention time window (the recommended setting). B and D were processed using Protover with a 10 min retention time window (+/−5 min) (the recommended setting). (TIF) [file pone.0094692.s002.tif]
