# Supplementary material for: Automated Protein Turnover Calculations from 15N Partial Metabolic Labeling LC/MS Shotgun Proteomics Data
Source: PLoS One. 2014 Apr 15;9(4):e94692. doi: 10.1371/journal.pone.0094692 (PMC3988089; doi:10.1371/journal.pone.0094692)
Supplement: Document S1 — Supplements. Detailed description of experimental procedures and methods, data analysis and comparison with other algorithm. Calculation of relative RIA, discussion of averaging scans as well as comparison of Retention Time settings. (DOC) [file pone.0094692.s003.doc]

# Supplements

## Plant Growth

The seeds of barrel medic (*M. truncatula* A17 cv. Jemalong) were surface sterilized and sown in pots containing a mixture of perlite: vermiculite 2:5 (v:v). Plants were grown under controlled conditions in a growth chamber (14h day and 10h night; 300 µmol m^–2^ s^–1^ photosynthetic photon flux density; 22°C day and 16°C night temperatures; 50–60% relative humidity). During the first week of growth, plants were watered with nutrient solution (Evans, 1981) containing 0.5mM ammonium nitrate. The following 6 weeks a nutrient solution with ammonium nitrate concentration of 2.5 mM was used for watering in order to enhance biomass accumulation and to keep plant growth performance identical during the initial developmental stage.

## ^15^N labeling experiment

Seven week old plants were randomly separated into two subsets: 1) control plants were further watered with ^14^N- ammonium nitrate fertilizer (2.5 mM), while 2) another set of plants was transferred to ^15^N- labeled ammonium nitrate (^15^N nitrate, 98% ^15^N; Sigma-Aldrich) containing growth medium. Plants were washed two times with water before medium application. Growth medium was supplied daily to pot capacity for 5 days. *M. truncatula* shoots were collected each day from the first day of ^15^N application, frozen in liquid nitrogen and stored to -80ºC until further processing.

## Protein Extraction

Three biological replicates were used for protein extraction. Two hundred mg of liquid nitrogen frozen material (fresh weight) were homogenized in 1 ml of urea buffer containing 50mM HEPES, pH 7.8 and 8 M Urea using a glass homogenizer. After centrifugation (10000g, 10min, 4°C), the urea-soluble proteins in the supernatant were precipitated overnight in five volumes of -20°C cold acetone containing 0.5% β-mercaptoethanol. The precipitate was pelleted at 4000g, 4°C for 15 min. The resulting pellet was washed with -20°C cold methanol containing 0.5% β-mercaptoethanol and again centrifuged (4000g, 4°C, 10 min). Air-dried protein pellets were dissolved in 800 µl of urea buffer (described above). Protein concentration was determined by Bradford assay using BSA as a standard.

## Protein Digestion

One hundred µg of protein was initially digested using 0.1 µg (1:1000, w/w) of endoproteinase LysC (Roche, Mannheim, Germany) during 5 h at 30°C. For the second digestion step, samples were diluted with trypsin buffer (10% Acetonitrile, 50mM Ammonium bicarbonate, 2 mM CaCl_2_) to a final concentration of 2 M Urea, and incubated overnight at 37°C with Poroszyme immobilized trypsin beads (3.3:100, v/w; Applied Biosystems, Darmstadt, Germany). The digest was desalted with C18-SPEC96-well plates (Varian, Darmstadt, Germany) according to the manufacturer’s instructions. The eluted peptides were vacuum-dried.

## nanoESI LC-MSMS

Peptide digests (2.5 µg each) were randomly applied to a RP column (Supelco Ascentis® Express Peptide ES-C18, 150x0.1mm) separated during a 90 min gradient ranging from 98% solvent A (0.1% FA in water, 2% ACN) to 80% solvent B (90% acetonitrile, 0.1% FA in water). For each treatment tree biological and two technical replicates were randomly analyzed to discriminate technical from biological variation. MS analyses were performed on a LTQ-Orbitrap XL (Thermo Fisher Scientific, Bremen, Germany), applying a top seven method. A full-scan range from 350 to 1,600 m/z was used. The resolution was set to 60,000. Dynamic exclusion settings were as described in [1]. Briefly, repeat count was set to one, repeat duration 20 s, exclusion list size 500, exclusion duration 60 s and exclusion mass width 10 ppm. Charge state screening was enabled with rejection of unassigned and 1+ charge states. Minimum signal threshold counts were set to 50,000.

## Peptide identification and generation of input list

The control (^14^N) as well as the treated (^15^N) samples resulted in a total of 15 files each (three biological replicates x five time points). Only the raw data files from non-labeled (^14^N) samples were processed and quantified using MaxQuant (v 1.4.0.3) with a combined protein fasta database (see 5.1, for MaxQuant details see 5.2).

## Medicago truncatula composite fasta generation

A composite protein-fasta-file was created by fusing the following three databases:

1.) Uniprot UniRef100 Medicago, origin: www.uniprot.org, Uniprot advanced-search Medicago truncatula (3880), UniRef100. The search was performed on May 7^th^ 2013. 54246 entries.

2.) IMGA, origin: http://medicago.org/genome/IMGAG/. 64123 entries.

3.) Database of Plant Ubiquitin Proteoasome System, origin: <http://bioinformatics.cau.edu.cn/plantsUPS/>. 1010 entries

4.) DCFI/MTGI/TC, origin: http://compbio.dfci.harvard.edu/tgi/cgi-bin/tgi/gimain.pl?gudb=medicago. Originally 412908 entries reduced to 59598 entries (after picking the longest continuous amino acid sequence).

The six-frame-translation was performed with an in-house tool. (The six-frame-translated longest-open-reading-frame protein-fasta-file contains six entries per accession number.) A new protein-fasta-file was created by picking only the longest continuous amino acid sequence per accession number.

The three fasta files described above were combined, producing a new fasta containing 131338 entries, which will be referred to as MT-fasta henceforth. Protein sequences 100% identical in sequence and in length were combined by subsequently adding one header after the other, separating them by the following characters " __***__ " (no matter if the redundancies originated from one or multiple fasta-files). All other entries were simply added to the new file. The first accession number of the header was repeatedly written to the very beginning of the header line, separated by a " | " (pipe character), in order to consistently view and parse the accession numbers.

## Peptide identification settings using MaxQuant

Trypsin (trypsin/P) was selected as the digestion enzyme, a maximum of two missed cleavages and no static or dynamic modifications were selected. Default settings were used except for the following modifications: “Global Parameters / Identification / Min. Peptides” was set to 2 and the “Parameters / Match between runs” box was selected. Within the “Group-specific parameters” the “Multiplicity” of 1 was selected, no variable modifications were selected, and for “Label-free quantification” LFQ was selected and “Fast LFQ” was deselected. Within the “Global parameters” no fixed modifications were selected. The default decoy database settings containing reversed sequences were used to estimate the false discovery rate (FDR) and a PSM as well as protein identification cutoff of FDR ≤ 1%.

## Generation of input list

We used a similar strategy to the Selective Peptide Extraction (SelPEx) [2] that allows for the targeted quantification of ^15^N-labeled peptides. The input list for *Medicago truncatula* shoots was generated from the MaxQuant data matrix derived from the ^14^N control samples (the “evidence” file). This list was filtered for target peptides according to the following criteria:

a) All peptide sequences were assigned a “+” in the “Reverse” or “Contaminant” column were disregarded.

b) Only peptide sequences with an “Intensity” value, a “Score” and a “PEP” score were retained.

This resulted in three separate lists of peptide identifications (A: 1055, B: 1118, and C: 916) which were combined and the duplicates removed. Finally, this resulted in a list of 1419 peptides. The peptide sequence, the dominant charge state (mean value of charge states rounded to the closest integer), as well as the recalibrated retention time (calculated by MaxQuant) were used to build the input list.

## Generation of mzML files

The proprietary “raw” file format (from Thermo Scientific™) is only legible under windows operating systems with the use of proprietary software. Thus the cross platform compatible, open standard mzML was used. All “raw” files were converted to “mzML” files using “msconvert” [3].

## Generation of LC/MS test data

To enable a rapid download and quick execution of the test data, the raw files were converted (analogous to 5.4 but) with the following restrictions. Limited to only MS1 scans, mz-range restricted to 402 to 866 mz, and retention time-range restricted from 28.85 to 32.3 min.

## Calculation of A_14_ and A_15_

Assuming that no enrichment of ^15^N has occurred at TP_0_ (first Time Point), the ratio of the monoisotopic peak to the intensity values of all other isotopic peaks of the spectral envelope of a peptide, reflect the experimentally derived intensity distribution of the natural isotopic composition. In order to differentiate the natural abundance from the enriched part (light and heavy) of an overlapping isotopic peak (see Figure 1 in Manuscript red and green species overlapping at e.g. the 5^th^ isotopic peak), the relative intensity values at TP_0_ are taken into account when calculating A_14_ and A_15_. At TP_0_ the sum of all intensity values produces A_14_, and A_15_ is set to Zero. For all other TPs, all intensity values $I_{\left[m/z\right]_{iTPn}}$of the isotopic peaks *i* occurring at TP_0_ are split and one part assigned to A_14_ and the rest to A_15_, depending on the intensity ratio $\frac{I_{\left[m/z\right]_{iTPn}}}{I_{\left[m/z\right]_{mTPn}}}$of the analogue isotopic peak *i* at TP0 to the monoisotope *m*. The calculation is as follows:

$A_{14}=\sum_{i=1}^{n_{TP0}} \frac{I_{\left[m/z\right]_{iTPn}}*I_{\left[m/z\right]_{iTP0}}}{I_{\left[m/z\right]_{mTP0}}}$,

$$A_{15}=\sum_{i=1}^{n_{TP0}} \left(I_{\left[m/z\right]_{iTPn}}-\frac{I_{\left[m/z\right]_{mTPn}}*I_{\left[m/z\right]_{iTP0}}}{I_{\left[m/z\right]_{mTP0}}}\right)$$

given that for each peak: $\frac{I_{\left[m/z\right]_{iTPn}}*I_{\left[m/z\right]_{iTP0}}}{I_{\left[m/z\right]_{mTP0}}}\leq I_{\left[m/z\right]_{iTPn}}$

the fractional part assigned to A_14_ is not greater than the intensity of the peak itself. Otherwise, A_14_ is simply the sum of all intensity values and no corresponding intensity is assigned to A_15_.

$$A_{14}=\sum_{i=1}^{n_{TP0}} I_{\left[m/z\right]_{iTPn}}$$

$$A_{15}=0$$

For all TPs, all intensity values $I_{\left[m/z\right]_{iTPn}}$ of isotopic peaks NOT occurring at TP_0_ are assigned to A_15_.

$$A_{15}=\sum_{i=nTP0+1}^{n_{TP0}} I_{\left[m/z\right]_{iTPi}}$$

## Post processing configuration options

The possibility of a negative effect of this filter strongly depends on the type of data used for analysis. If the experimenter realizes that this filter is too stringent for the data at hand, he/she could simply change a single line of code (from „RIAincreasing = True” to „RIAincreasing = False” in „run_experimentfile.py“), thus stopping the application of this filter.

## Averaging over multiple scans vs. one single scan

Chromatographic peak widths are not constant for all analytes, can vary between replicates and are dependent on analyte concentration and chromatographic conditions. Thus “fronting” and or “tailing”, very narrow as well as broad peaks, and peaks with multiple shoulders are commonly observed in liquid chromatography of highly complex samples. Determining the beginning and end of an eluting substance is a challenging task by itself. Averaging over multiple scans in order to produce one single spectrum necessitates the additional step of binning in the mass (m/z) dimension (a simple arithmetic mean will certainly not suffice, since the intensities of almost identical m/z values would not be summed). Also, the fact that the mass accuracy and relative isotope abundances increase with the signal intensity needs to be considered for such an endeavor. A reliable and fast averaging method could lead to smoother spectral envelopes and a better signal to noise ratio in comparison to single scans, but could also raise the complexity of the spectrum or lead to overlaps of isotopic peaks from different analytes that would not occur in a single scan. In a nutshell, there are advantages and disadvantages for both approaches. The utilization of a single scan is straightforward, but averaging over multiple scans isn’t. The presented algorithm is trained to pick the best possible scan within the user-given retention time range, coping with large retention time deviations that can occur in high throughput studies. Additionally, the functionality of the presented program could easily be extended with a proper algorithm (if available) that averages over multiple spectra.

## Comparison of Retention Time settings

Figure S1.Ai, Aii, and Aiii illustrate how the individual RIA values of peptides attributed to the same relatively high turnover protein are drawn closer together when applying smaller Retention time settings and that the best results are achieved with individually optimized Retention time window settings for each peptide for each file (for the data presented in Figure S1.Aiii and Biii this means 140 manual data entries for the beginning and the end of the retention time (14 peptides * 5 LC/MS measurements * 2 beginning/ending Rt). The analogue processing steps were performed for a relatively low turnover protein, see Figure S1.Bi, Bii, and Biii. In the latter plots, the values are less stringent, probably due to the fact that low protein turnover produces low intensity heavy isotope envelopes, which are hard to differentiate from noise when averaging over multiple scans.

## References

1. Hoehenwarter W, Wienkoop S (2010) Spectral counting robust on high mass accuracy mass spectrometers. Rapid Commun Mass Spectrom: 3609–3614. doi:10.1002/rcm.

2. Castillejo MA, Staudinger C, Egelhofer V, Wienkoop S (2014) Medicago truncatula proteomics for systems biology: novel rapid shotgun LC-MS approach for relative quantification based on Full-Scan Selective Peptide Extraction (Selpex). Plant Proteomics Methods Mol Biol 1072: 303–313.

3. Chambers MC, Maclean B, Burke R, Amodei D, Ruderman DL, et al. (2012) A cross-platform toolkit for mass spectrometry and proteomics. Nat Biotechnol 30: 918–920. doi:10.1038/nbt.2377.
